# The Light Chain Allosterically Enhances the Protease Activity of Murine Urokinase-Type Plasminogen Activator

**DOI:** 10.1021/acs.biochem.4c00071

**Published:** 2024-05-23

**Authors:** Constanza Torres-Paris, Harriet J. Song, Felipe Engelberger, César A. Ramírez-Sarmiento, Elizabeth A. Komives

**Affiliations:** † Department of Chemistry and Biochemistry, Mail Code 0309, University of California San Diego, 9325 S Scholars Dr, La Jolla, California 92161, United States; ‡ Institute for Biological and Medical Engineering, Schools of Engineering, Medicine and Biological Sciences, Pontificia Universidad Católica de Chile, Santiago 7820436, Chile; § ANID - Millennium Science Initiative Program - Millennium Institute for Integrative Biology (iBio), Santiago 8331150, Chile

## Abstract

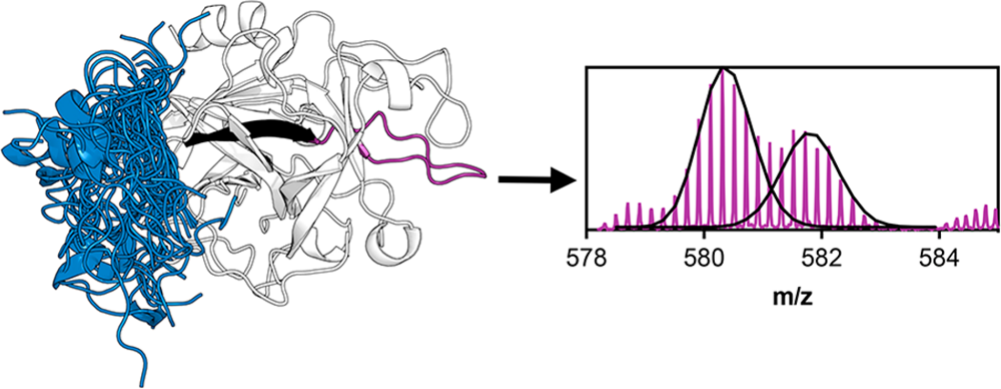

The active form of the murine urokinase-type plasminogen activator (muPA) is formed by a 27-residue disordered light chain connecting the amino-terminal fragment (ATF) with the serine protease domain. The two chains are tethered by a disulfide bond between C1_CT_ in the disordered light chain and C122_CT_ in the protease domain. Previous work showed that the presence of the disordered light chain affected the inhibition of the protease domain by antibodies. Here we show that the disordered light chain induced a 3.7-fold increase in *k*_cat_ of the protease domain of muPA. In addition, hydrogen–deuterium exchange mass spectrometry (HDX-MS) and accelerated molecular dynamics (AMD) were performed to identify the interactions between the disordered light chain and the protease domain. HDX-MS revealed that the light chain is contacting the 110s, the turn between the β10- and β11-strand, and the β7-strand. A reduction in deuterium uptake was also observed in the activation loop, the 140s loop and the 220s loop, which forms the S1-specificty pocket where the substrate binds. These loops are further away from where the light chain seems to be interacting with the protease domain. Our results suggest that the light chain most likely increases the activity of muPA by allosterically favoring conformations in which the specificity pocket is formed. We propose a model by which the allostery would be transmitted through the β-strands of the β-barrels to the loops on the other side of the protease domain.

## Introduction

**Figure 1:**
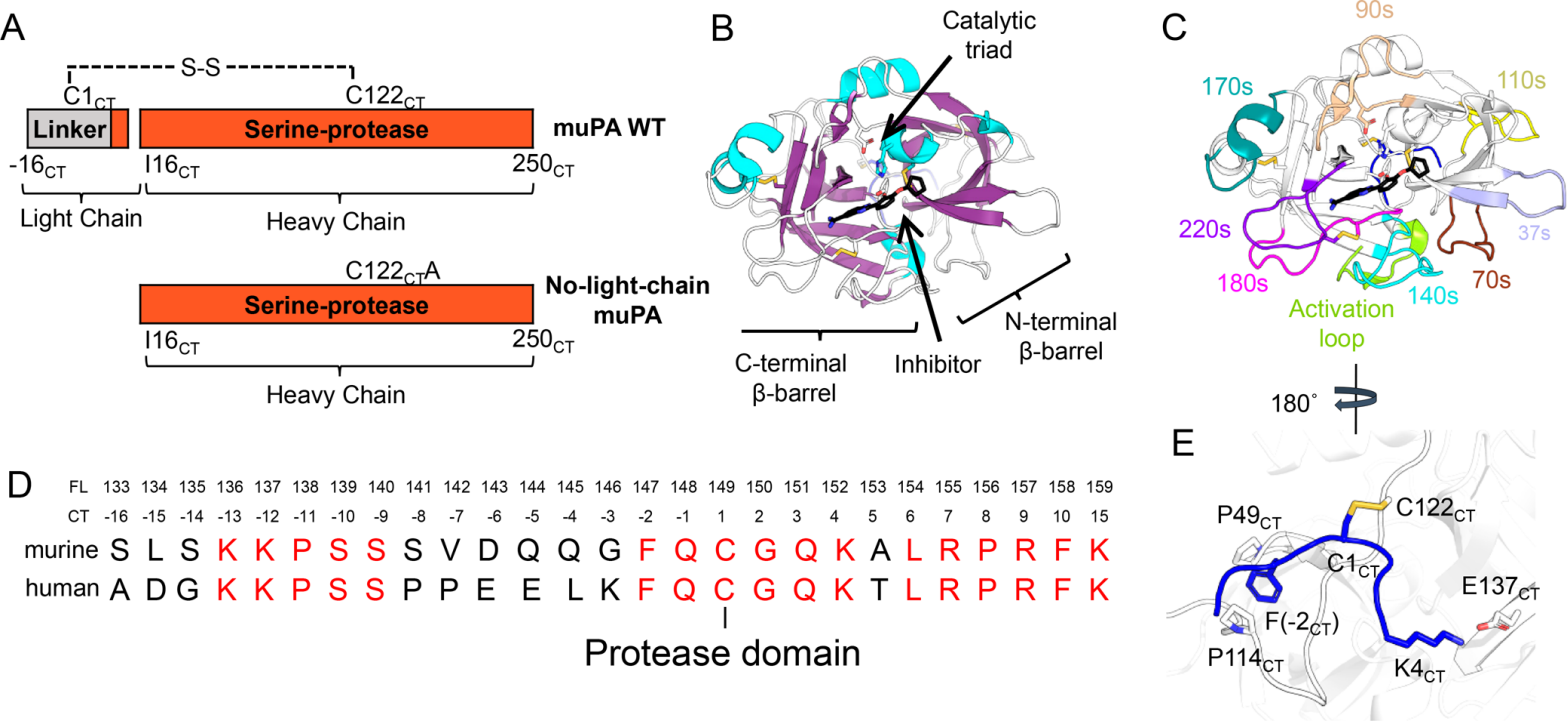
Structure of muPA. A. Schematics of the proteins used in this study. B. Canonical view of the crystal structure of the protease domain of human uPA (huPA) containing part of the light chain (PDB: 1O3P). The N-terminal β-barrel contains six β-strands (β1- β6) and the C-terminal β-barrel contains another six (β7- β12). The catalytic triad residues H206 (57_CT_), D257 (102_CT_) and S358 (195_CT_) (shown as sticks) are in the interface between the two β-barrels. An active site covalent inhibitor is shown in black sticks. The structure is colored according to the secondary structure. C. Canonical view of the crystal structure of huPA showing the loops and their names. D. The sequence of the disordered region of the light chain of murine uPA (muPA) and huPA. The numbering of each residue in the full-length (FL) and chymotrypsin-like (CT) numbering. The conserved residues are shown in red. The side chain of C149 (1_CT_) makes a disulfide bond with the protease domain. E. Zoomed view of the rotated view of the crystal structure of huPA (PDB: 1O3P) showing the residues in the light chain (blue backbone) and the protease (white backbone) that have been proposed as interactions. All the residue numbers correspond to the chymotrypsin numbering. Here we show the huPA structures because no muPA structures are available with the light chain.

The murine urokinase-type plasminogen activator (muPA, UniProt P06869) is a serine protease involved in fibrinolysis, cell adhesion, cell migration and tissue remodeling.^1^ This enzyme catalyzes the extracellular activation of the zymogen plasminogen into its active form, plasmin. In addition to its serine protease domain, muPA also has an N-terminal fragment (ATF) which consists of an epidermal growth factor (EGF) domain, a Kringle domain and a disordered linker between the Kringle domain and the serine protease domain (Figure 1). These additional domains have been associated with binding to other proteins like the muPA receptor (uPAR) and some integrins.^2−5^ The muPA is a highly disulfide-bonded protein which contains a total of 12 disulfide bonds: three in the EGF, three in the Kringle, five in the serine protease and one connecting the serine protease and the disorder linker.

As most serine proteases, muPA is synthesized as an inactive single-chain zymogen which has all its disulfide bonds already formed, but still needs to be cleaved in the disordered linker between K159 (15_CT_) and I160 (16_CT_) to become an active protease.^6^ Note that it is traditional to number the residues of the serine protease domain in two ways: in its full-length numbering (no subscript) and in comparison to their structural homologue, chymotrypsin (CT subscript). After cleavage/activation, the new N-terminus of I160 (16_CT_) inserts into the activation pocket, forming a salt bridge with D357 (194_CT_), which is next to the catalytic S358 (195_CT_) and orients the serine side chain into the correct conformation for catalysis.^7,8^ The disordered linker remains attached to the protease domain by way of the disulfide bond between C149 (1_CT_) in what is termed the light chain and C281 (122_CT_) in what is termed the heavy chain or protease domain (Figure 1A).

The full-length muPA has not been crystallized likely due to the disordered segment connecting the ATF and the protease domain. However, the structures of the human ATF and the human and the murine protease domain have been solved (Figure 1 B–C).^7−9^ The protease domain of muPA has always been crystallized in the absence of the light chain. The crystal structure of human uPA (huPA, PDB: 1O3P) shows electron density for only eight residues of the 27 that comprise the light chain (Figure 1 E), from L(−4_CT_) to K4_CT_, most of which are conserved between muPA and huPA (Figure 1 D). The structure of muPA without the light chain crystallized with two different conformations of the protease domain, one of which corresponded to a nonchymotrypsin-like fold.^8^ This structure showed a misaligned catalytic triad and an antiparallel-to-parallel transition of the β9 strand in the C-terminal β-barrel, which extends the 180s and 170s loops and disorganizes the specificity pocket.

In addition to the structural features, dynamic allostery also regulates the activity of serine proteases.^10,11^ Hydrogen–deuterium exchange mass spectrometry (HDX-MS) has been extensively used to characterize the changes in dynamics of serine proteases in solution.^12−16^ Kromann-Hansen et al. suggested that the canonical chymotrypsin-like fold and this alternate fold may be in equilibrium and used HDX-MS to show that the nonchymotrypsin fold had much higher deuterium incorporation in the 170s, 180s and 220s loops.^8^ These authors were able to isolate monoclonal antibodies that stabilized the chymotrypsin-like fold,^8,10,17,18^ and could inhibit or stimulate the activity of muPA^8,10^ suggesting that the different antibodies could shift the equilibrium between two conformational ensembles with different enzymatic activities. An antibody that stimulated activity reduced the deuterium exchange in the 170s, 180s and 220s loops, and an antibody that inhibited activity increased it. Therefore, activity and dynamics of certain loops in muPA seem to be inversely related.

Kromann-Hansen et al. showed that the inhibition capacity of an antibody was significantly lower in the presence of the disordered portion of the light chain compared to just the heavy chain of muPA,^17^ suggesting that the light chain was changing the equilibrium of conformations of the protease domain in the antibody binding site.^17^ In addition, the double mutation of K152(4_CT_)G and F147(−2_CT_)A (K4_CT_G and F(−2)_CT_A from here on) was enough to change the inhibitory capacity of the antibody toward muPA to the same levels as if there was no light chain present at all.^17^ These authors did not report whether the presence or absence of the light chain affected the activity of the protease domain, but it is known that some light chain mutations in thrombin cause severe bleeding phenotypes^19−22^ and *in vitro* studies have confirmed that some of these mutations affect the catalytic activity of thrombin.^23−25^

In this work, we studied the effect of the light chain and its mutations K4_CT_G and F(−2_CT_)A on the activity and dynamics of the protease domain of muPA. Our results show that the presence of the light chain significantly increases the catalytic rate of muPA and that the K4_CT_G and F(−2_CT_)A mutations provoke the partial and total loss of that increase in activity, respectively. HDX-MS revealed where the light chain interacts with the protease domain and where its effects are felt allosterically in the specificity pocket and the catalytic triad. Our results support a model in which allostery is transmitted from the contact points with the light chain to the catalytically relevant regions of the protein through the β-strands in muPA.

## Materials and Methods

### Variants of muPA

Two variants of muPA (Uniprot: P06869) were purified. The first one consisted of residues 133 (−15_CT_) to 413 (250_CT_) which contained the disordered part of the light chain, the protease domain, an N-terminal 6xHis-tag and a TEV protease cleavage site. After plasmin cleavage, the two-chains on this protein remain together. This first protein will be called “muPA WT” from here on. The other muPA variant used in this study consisted of residues 150 (2_CT_) to 413 (250_CT_) which contained only the heavy chain of the protease domain of muPA, a short segment of the disordered linker, the C281(122_CT_)A mutation so that the light chain cannot disulfide bond to the protease domain, and an N-terminal 6xHis-tag. Plasmin cleavage of this construct between K159 (15_CT_) after I160 (16_CT_) would release the heavy chain of muPA which is not disulfide bonded due to the C281(122_CT_)A mutation. This processed active enzyme will be called “No-Light-Chain muPA” from here on. The mutations F(−2_CT_)A and K4_CT_G were carried out by site-directed mutagenesis of the muPA WT gene as a template, using the DpnI method.

### Expression and Purification of muPA

The expression of muPA in inclusion bodies, its refolding and its purification were carried out as described previously.^8,16^ The protein concentration was measured by absorbance at 280 nm, using a Nanodrop. The extinction coefficients at 280 nm of muPA WT were 47620 M^–1^cm^–1^, when all cysteines were oxidized, and 46870 M^–1^cm^–1^ when all cysteines were reduced. The extinction coefficient at 280 nm of No-light-chain muPA was 46005 M^–1^cm^–1^, when all cysteines were oxidized, and 45380 M^–1^cm^–1^ when all cysteines were reduced.

### Amidolytic Activity Assay

The activity of muPA was measured by preincubating with 0.25 mM – 6 mM of the chromogenic substrate pyro-Glu-Gly-Arg-pNA or DPG444–25 (Diapharma, West Chester, OH) (also referred to as S2444) in 200 μL of Phosphate-buffered Saline (PBS, 10 mM Na_2_HPO_4_, 1.8 mM KH_2_PO_4_, 2.7 mM KCl, 137 mM NaCl, pH 7.4) in a 96-well microplate for 10 min at 37 °C, before adding 10 nM of muPA (preincubated at 37 °C) to start the reaction. The reaction was monitored for 10 min at 37 °C and the absorbance at 405 nm (A_405 nm_) was measured every 20 s. Initial rates were calculated using data from only the first 5 min. Reactions were run in duplicate.

### Hydrogen–Deuterium Exchange Mass Spectrometry

HDX-MS was performed as previously described.^16^ The H_2_O buffer was PBS pH 7.4, which was lyophilized and resuspended in deuterium oxide (D_2_O) to prepare the D_2_O buffer. Four μL of protein (WT 20 μM; No Light Chain 10 μM; F(−2)_CT_A 8.5 μM; K4_CT_G 13 μM) were incubated for 5 min at 22 °C and then mixed with 56 μL of H_2_O Buffer as a control or D_2_O buffer for deuteration times of 0, 30 s, 60 s, 120 s, 600 s at the same temperature. The reaction was quenched with 60 μL of 250 mM TCEP, pH 2.5 at 0 °C. Mass spectrometry was carried out using a Waters Synapt G2Si system with HDX technology (Waters Corporation) using the same methods and parameters as previously reported.^16^ Peptides were identified using PLGS 2.5 (Waters Corporation), and analyzed using DynamX (Waters) and DECA as previously described.^26^ Bimodal distributions of hydrogen–deuterium exchange were analyzed and deconvoluted using HX-Express3.^27,28^

### Modeling and Accelerated Molecular Dynamics (AMD)

The atomic coordinates of No-Light-chain muPA were obtained from the crystal structure (PDB: 5LHR) from which the bound active site nanobody was removed. Modeling of muPA WT was carried out by docking the 27 residues of the light-chain to the crystal structure of the protease domain of muPA (PDB: 5LHR) without the nanobody, using Rosetta FlexPepDock^29^ with harmonic restraints to conserve the salt bridge between K4_CT_ and E296 (137_CT_) and the hydrophobic interactions between F(−2_CT_), P198 (49_CT_) and P273 (114_CT_).^17^ The disulfide bond was added using VMD.^30^ The dynamics of muPA WT and No Light Chain muPA were simulated by performing AMD using the AMBER16 simulation suite as described previously,^31^ with two exceptions: 1) the simulation cell was defined such as that the distance between the edge of the simulation box and the surface of the solute was at least 14 Å, and 2) the initial prerun conventional MD (CMD) was 250 ns. The two AMD simulations were performed for 750,000,000 steps with a time step of 2 fs for each protein, which is equivalent to a 1.5 μs CMD simulation. These simulations were run in duplicates. Clustering was performed using the k-means algorithm implemented in CPPTRAJ^32^ to generate a representative set of 20 structures.

## Results

### Effect of the Light Chain on the Amidolytic Activity of muPA

To evaluate the effect of the disordered region of the light chain on the catalytic activity of muPA, we purified, refolded and plasmin-activated muPA WT (residues 133–413; −15_CT_-250_CT_) and No-light-chain uPA (residues 160–413; 1_CT_-250_CT_) and measured their catalytic activity using the chromogenic substrate analog, S2444 (Table 1, Figure S1). The presence of the light chain induced a 3.7-fold increase in *k*_*cat*_, without significantly altering the K_M_.

**Table 1:** Catalytic Constants of the Amidolytic Activity of muPA Variants

	*K*_M_ (mM)	*k*_cat_ (s^–1^)	*k*_cat_/*K*_M_ (s^–1^ M^–1^)
WT	1.0 ± 0.2	52 ± 4	5.2 (±1.1) × 10^4^
No-light-chain	1.4 ± 0.6	14 ± 2	1.0 (±0.5) × 10^4^
F(−2_CT_)A	1.4 ± 0.6	12 ± 2	0.9 (±0.4) × 10^4^
K4_CT_G	0.7 ± 0.2	22 ± 2	3.1 (±0.9) × 10^4^

Previous authors^17^ had proposed that the light-chain would interact with the protease domain via a salt bridge between K4_CT_ in the light chain and E296 (137_CT_) in the protease domain, and by hydrophobic interactions between F(−2_CT_) in the light chain and P198 (49_CT_) and P273 (114_CT_) in the protease domain (Figure 1 E).^17^ To test whether these light chain residues were important for protease catalysis, we generated the F(−2_CT_)A and K4_CT_G mutants. These mutant proteins were purified and refolded and their amidolytic activity was tested (Table 1, Figure S1). The K4_CT_G mutation decreased the *k*_*cat*_ of muPA by 2-fold and the F(−2_CT_)A mutation decreased it by 4-fold, to the same level as the protease domain without the light chain. Neither mutation affected the K_M_ significantly.

### Interaction of the Light Chain with the Region Where It Is Connected to the Protease Domain by a Disulfide Bond

**Figure 2:**
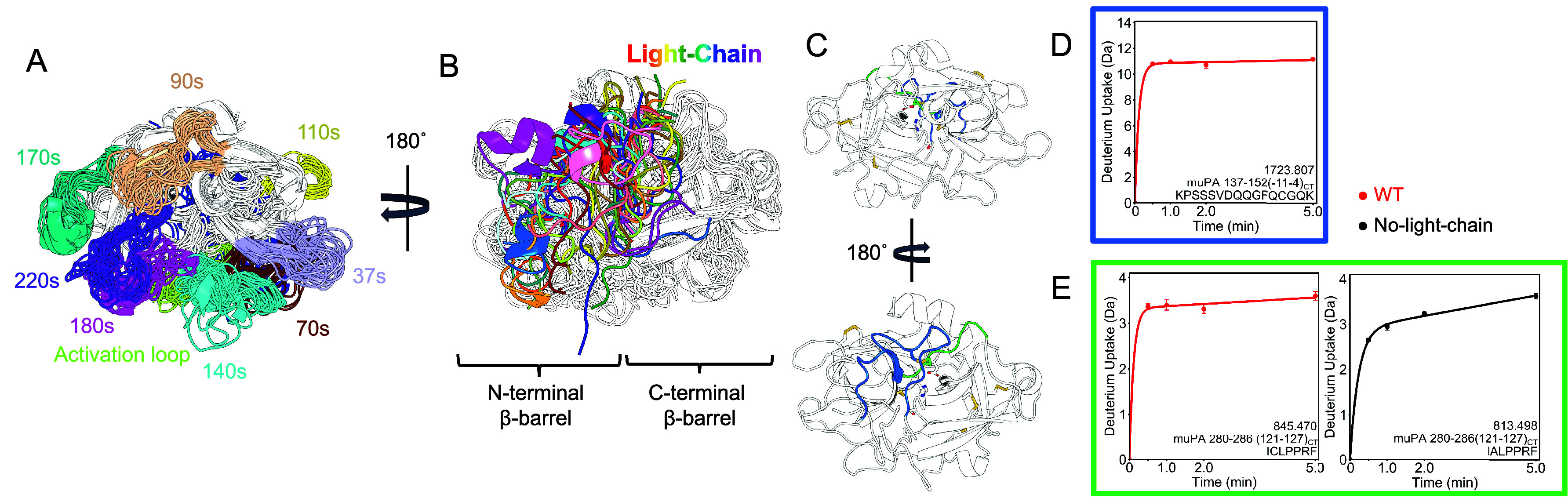
Disordered light chain is very dynamic and does not appear to adopt a defined position on the protease domain. A. Canonical view into the active site of the ensemble of conformations acquired by muPA WT during AMD. The loops are colored in the same color scheme as the crystal structure of human uPA in Figure 1C. B. 180° rotated view of muPA WT showing the light chain ensemble of conformations acquired by AMD. Each light chain conformation is colored in a unique shade. C. Canonical (top) and rotated (bottom) view of a conformation from the ensemble of muPA WT showing the light chain colored in blue with residues 280–286 (121_CT_ −127_CT_) colored in green. The catalytic triad residues and the disulfide bonds are shown as sticks, and the inhibitor is colored black. D. Deuterium uptake plot of the light chain peptide of muPA WT. Each data point represents the average of three technical replicates and the error bars represent the standard deviation. E. Deuterium uptake plots of the protease peptides which contain either C122 (left) or C122A (right), the site where the light chain covalently binds to the protease.

To understand how the light chain was affecting the protease domain of muPA, AMD and HDX-MS were carried out to predict where the light chain was interacting with the protease domain. AMD revealed that the light chain is highly dynamic and does not have a single preferred conformation in the ensemble (Figure 2 A, B). HDX-MS was performed to map the interactions between the light chain and the protease domain in solution (Table S1). In agreement with the AMD, the peptides covering the light chain were highly deuterated even after 30 s, as expected for a disordered region (Figure 2 D).

Interestingly, the amides in the protease domain near the disulfide bond that connects the protease domain with the light chain (C281(122_CT_)) were also highly deuterated (Figure 2 E), which suggested that these residues were mostly solvent exposed and were not interacting with the light chain strongly enough to prevent deuterium exchange of this surface region. Very similar trends were observed for the light chain mutants (Figure S2).

### Effect of the Light Chain on the Activation Loop

**Figure 3:**
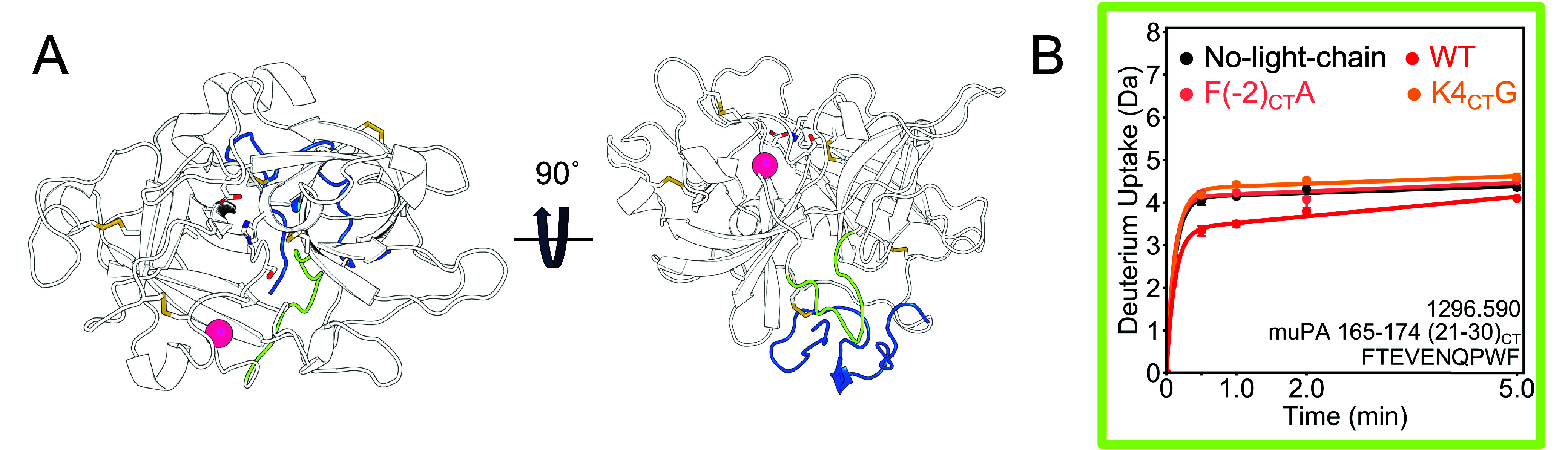
Effect of the light-chain on the activation loop. A. The activation loop is located downstream of the new N-terminus (fuchsia sphere), at the bottom of the protease domain if looked from the front (left). Residues 165–174 (21_CT_-30_CT_)) are part of the activation loop and are colored green. The light chain is shown in blue. The catalytic triad residues and the disulfide bonds are shown as sticks. B. Deuterium uptake plot of residues 165–174 (21_CT_-30_CT_) in the activation loop.

The amide exchange in regions of the protease domain that are known to contribute to catalysis was analyzed. First, we sought to analyze the new N-terminus of I160 (16_CT_) which inserts into the activation pocket, where it interacts with D357 (194_CT_) adjacent to the catalytic serine, S358 (195_CT_).^8^ Although no peptides containing the new N-terminus were observed in the HDX-MS experiment, some peptides representing the activation loop, which immediately follows the new N-terminus and connects it to the N-terminal β barrel were observed (Figure 3, green). The deuterium uptake of this region was lower in the presence of the WT light chain, whereas uPA missing the light chain as well as the F(−2_CT_)A and the K4_CT_G mutations showed higher deuterium uptake. A less dynamic activation loop is reflective of a less dynamic new N-terminus which would adopt the correct orientation for catalysis more often.

### Effect of the Light Chain on the N-Terminal β-Barrel

**Figure 4:**
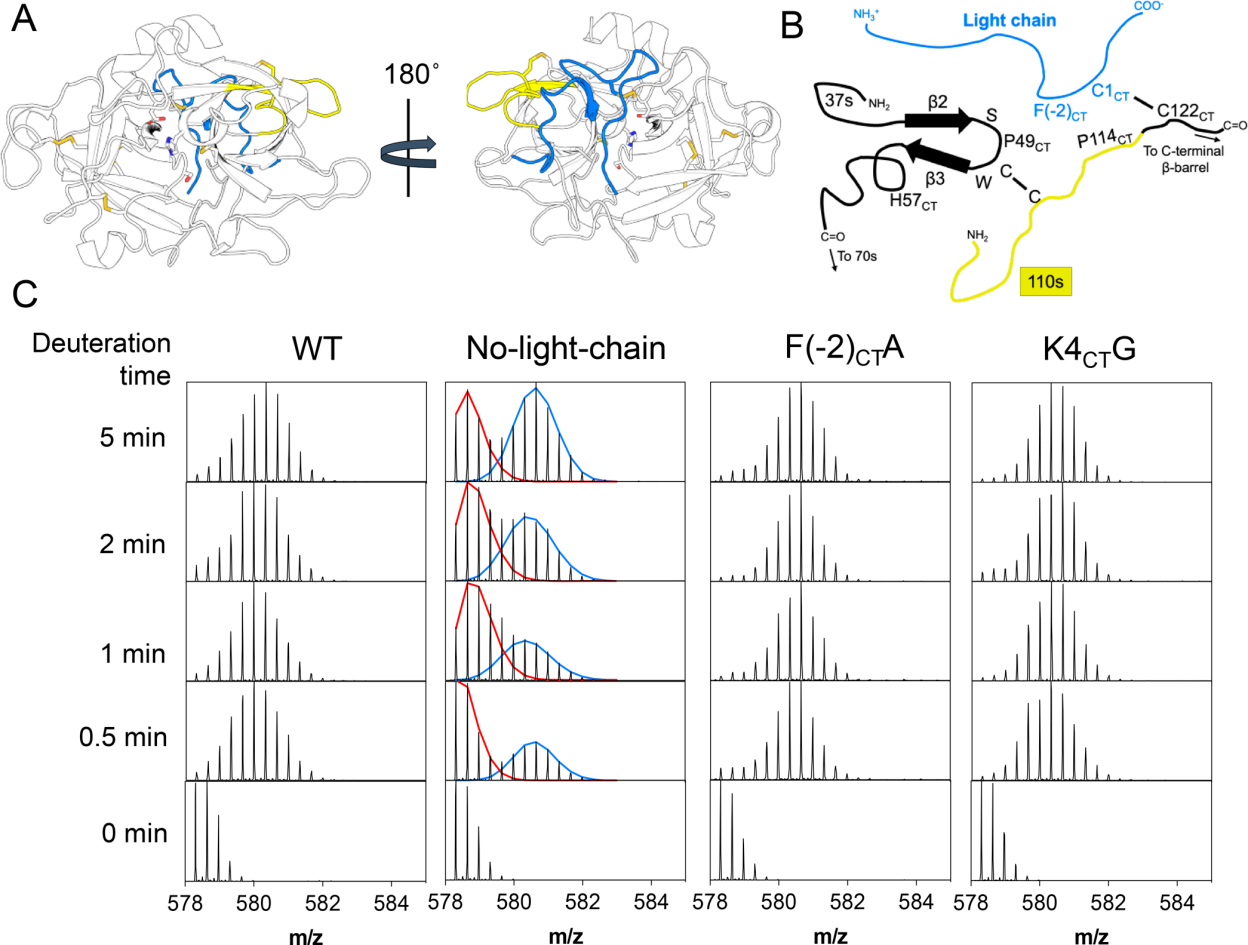
Effect of the light chain on the 110s loop. A. A conformation from the AMD ensemble of muPA WT shows that the light chain contacts the N-terminal β-barrel around P198 (49_CT_), in the turn between β2 and β3, and P273 (114_CT_) located in the connection between the two β-barrels and the 110s loop. Residues 261–276 (106_CT_-117_CT_) covering the 110s loop and part of the connection between the two β-barrels is shown in yellow. The light chain is colored in blue. B. Cartoon representation of the interactions between the light chain and the N-terminal β-barrel. C. Deuterium incorporation spectra of peptide 261–276 (106_CT_-117_CT_) sequence LKIRTSTGQCAQPSRS, in the +3 charge state. The *x*-axis of each plot is *m*/*z* and the *y* axis is the intensity at each peak. Each column represents one muPA variant (WT, no light chain, F(−2_CT_)A, K4_CT_G), and each row is a deuteration time point (0–5 min). This region of the protease shows a bimodal deuterium uptake distribution in the protease that is missing the light chain. The envelope of the higher m/conformation is shown in blue, and the one for the lower *m*/*z*, in red.

The crystal structure of huPA shows that F(−2_CT_) in the light chain interacts with a hydrophobic pocket formed by P198 (49_CT_) and P273 (114_CT_) in the N-terminal β-barrel (Figure 1 E)^17^. While P198 (49_CT_) is located in the turn between β2 and β3, P273 (114_CT_) is in the connector between the two β-barrels (Figure 4 A, B). Our HDX-MS data identified a peptide containing P273 (114_CT_) that showed bimodal deuterium uptake only when the light chain was not present (Figure 4 C). One of the states corresponds to a solvent exposed state (higher *m*/*z*, blue) and the other to a more protected state (lower *m*/*z*, red). The proportion of the more protected state decreased over time, and the proportion of the solvent-exposed state increased over time suggesting that the two states follow EX1 kinetics and exchange on a slow time scale relative to that of the HDX-MS experiment.^33^ The more protected state is present only in the absence of the light chain. We interpret this more protected state as a collapse of residues A271 (112_CT_), Q272 (113_CT_) and P273 (114_CT_) contained within this peptide into the hydrophobic pocket that is normally formed by F(−2_CT_), P198 (49_CT_) and P273 (114_CT_) but can also be formed by the F(−2_CT_)A mutant.

### Effect of the Light Chain on the Catalytic Triad and the Specificity Pocket

We also examined the peptides containing the catalytic triad residues: H206 (57_CT_), D257 (102_CT_) and S358 (195_CT_). Only one peptide containing H206 (57_CT_) was detected (Figure S3 A-C). Mutation of F(−2_CT_) or deletion of the light chain did not affect the deuterium uptake of this region, however, the K4_CT_G mutation caused this region to exchange more. This peptide also contains the turn between β2 and β3, which includes P198 (49_CT_) that is shown in the crystal structure of huPA to interact with the light chain (Figure 1 E). Upstream of β2 is the 37s loop and downstream of β3 is the 70s loop, both of which have slightly decreased deuterium uptake only in the presence of the WT light chain (Figure S3 D, E). This slight decrease could be the result of either some transient interactions with the light chain or of allostery as a consequence of the interaction of the light chain with the turn between β2 and β3 that may be transferred through the β2 and β3 strands.

Several peptides were detected that contain the catalytic D257 (102_CT_) located at the bottom of the 90s loop in the N-terminal β-barrel (Figure S4). The crystal structure of huPA shows that the 90s loop is longer than in other serine proteases and that it could be interacting with the 170s loop (Figure S4 B). HDX-MS revealed that both the 170s loop and the first part of the 90s loop show lower deuterium uptake when the light chain is present compared to the No-light-chain protein (Figure S4 C–D). The last part of the 90s loop is completely buried and does not show significant deuterium uptake in any condition (Figure S4 E), which suggests that all the amides are stably hydrogen bonded. This part of the 90s loop is connected to the β6 strand in the N-terminal β-barrel, which connects to the 110s loop. This β6 strand has been shown by NMR to transmit the allostery in thrombin from the thrombomodulin binding site to the catalytic triad.^34^ The authors of that study were unable to see changes in HDX-MS in this region but were able to detect the dynamic motions by NMR. Our results suggest that the light chain could be interacting with the back of the 110s loop and the β6 strand could be transmitting that interaction and affecting the dynamics of the rest of the 90s loop and the 170s loop.

**Figure 5:**
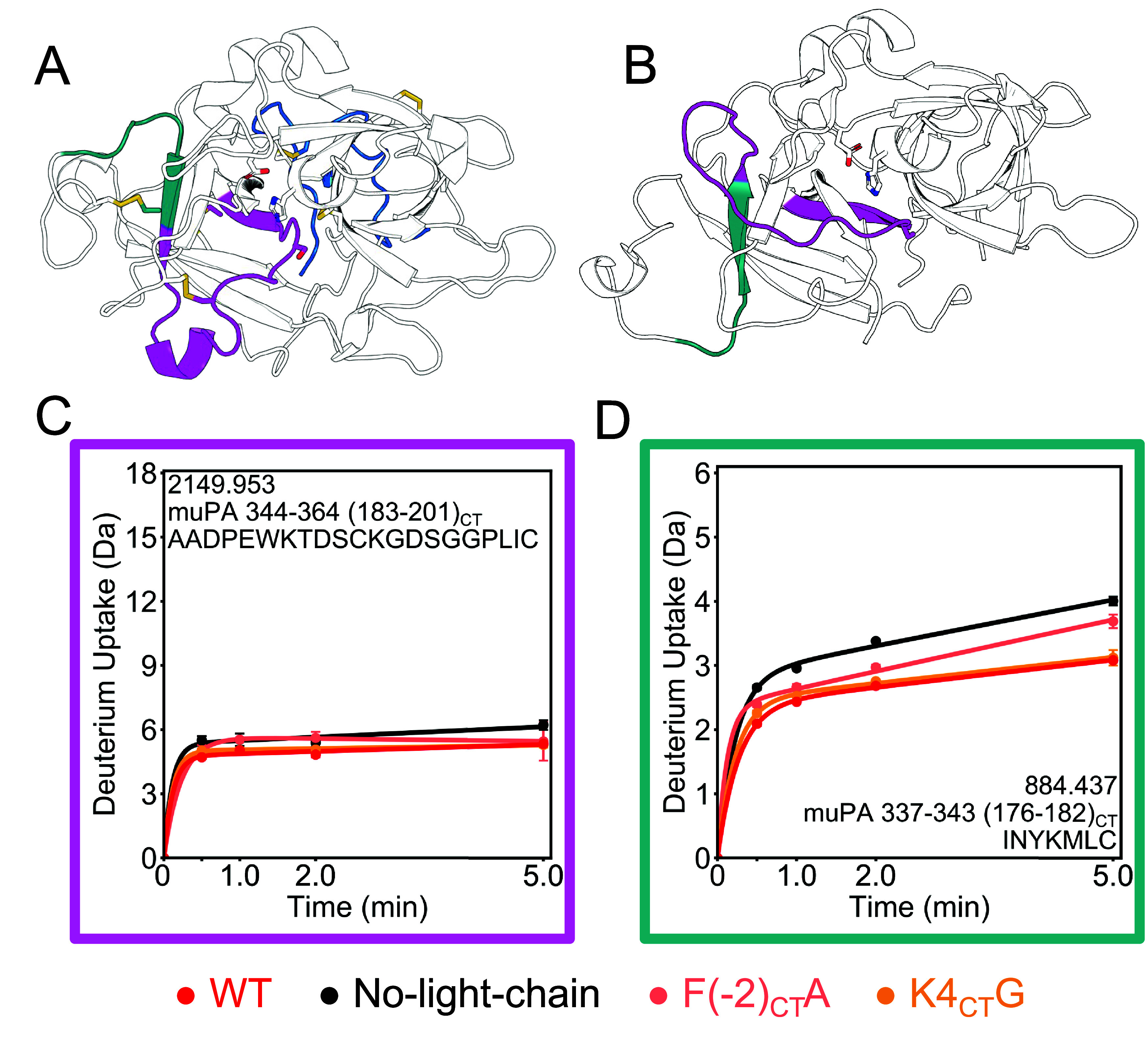
Light chain reduces the dynamics of the β9 strand and does not affect the 180s loop. A. A representative conformation of muPA WT from the AMD simulation shows the β9 strand (teal) and the 180s loop (magenta), which are in the C-terminal β-barrel. The catalytic triad residues are shown as sticks. B. The non-chymotrypsin-like fold of muPA No-light-chain (PDB: 5LHS) has a distorted C-terminal β-barrel. C. Deuterium uptake plot of residues 344−364 (183CT-201CT) in the 180s loop. This peptide encompasses the catalytic S358(195CT). D. Deuterium uptake plot of residues 337−343 (176CT-182CT) in the β9 strand.

The catalytic serine, S358 (195_CT_) is in the 180s loop (Figure 5 A, magenta). The dynamics of this loop slightly decreased in the presence of the light chain (Figure 5 C). Previous authors^8^ had shown that the protease domain of muPA without the light chain could also acquire a nonchymotrypsin-like conformation (Figure 5 B) where the β9 strand (teal), connecting the 170s and 180s loops, was upside down and the 170s and 180s loops were extended, by comparison to the canonical chymotrypsin-fold of muPA (Figure 1 B and C).

The authors discussed that only the chymotrypsin-like fold was active because only in this conformation the catalytic triad is aligned, the substrate binding pocket is formed, and the new N-terminus is inserted near the catalytic triad. The authors also showed that the binding of some nanobodies or an active site inhibitor would shift the equilibrium of conformations favoring the chymotrypsin-like conformation.^8,10^ Our results show that the light chain could also be favoring the chymotrypsin-like fold in muPA, as the β9 strand shows reduced deuterium exchange in the presence of the light chain (Figure 5 D). The K4_CT_G mutation did not affect the deuterium uptake of the β9 strand and the F(−2_CT_)A mutation caused the β9 strand to be in an intermediate state between the WT and the No-light-chain conformation (Figure 5 D).

**Figure 6:**
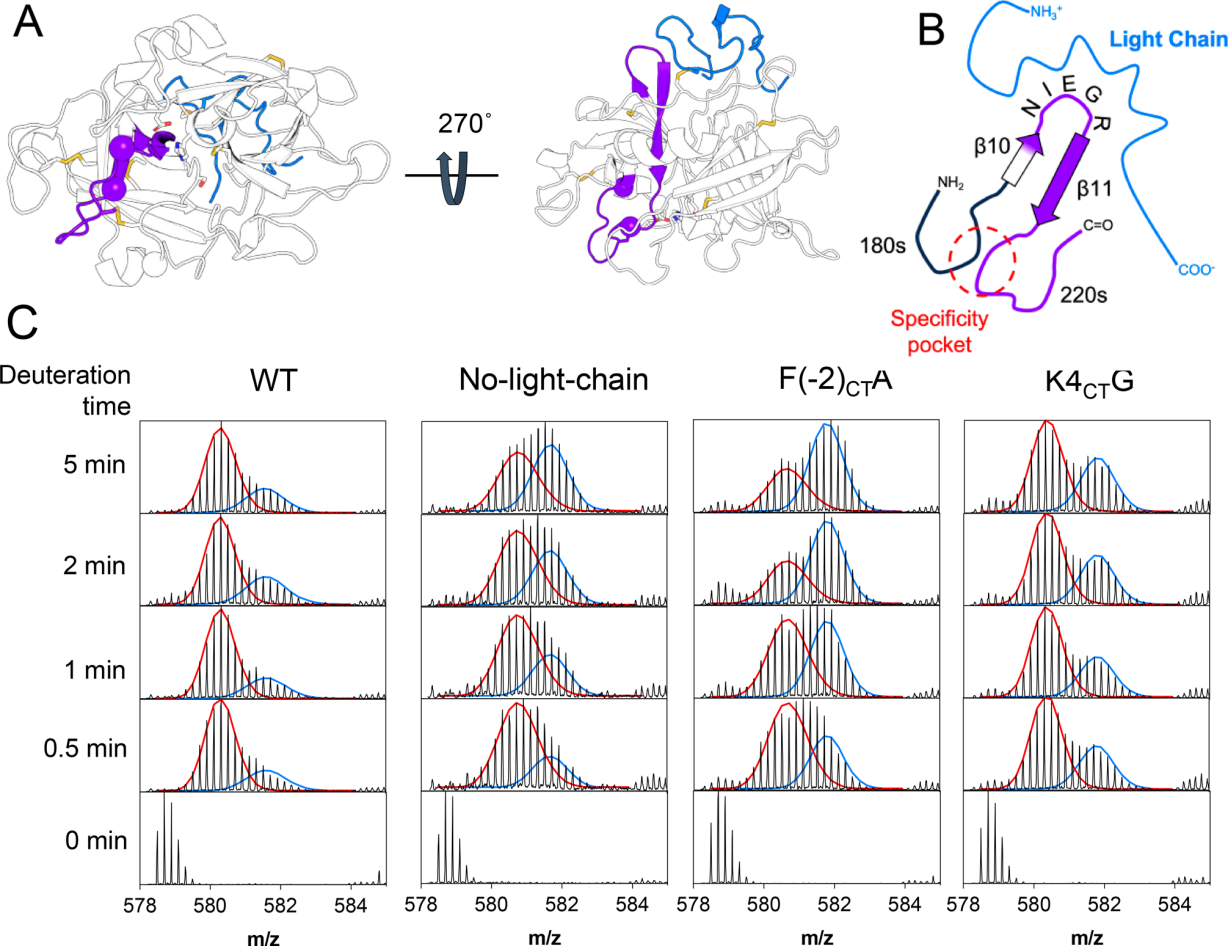
Light chain affects the dynamics of the 220s loop and the β10-β11 turn. A. The 220s loop (purple) is in the C-terminal β-barrel of muPA WT. The S1 specificity pocket is formed by two residues in the 220s loop (Gly 379 (216_CT_) and Gly 389 (226_CT_), shown as purple spheres) and a residue in the 180s loop (Asp 352 (189_CT_), shown as a white sphere. The structure in the canonical view of the protein and the structure rotated by 270° on the *x* axis are shown for comparison. The light-chain is shown in blue, and the peptide analyzed by HDX-MS corresponding to residues 365–391 (202_CT_-228_CT_), covering part of the 220s loop, β10 and β11 strands and the turn between those β strands, is shown in purple. B. Cartoon model showing how the light chain would interact with the region corresponding to residues 365–391 (202_CT_-228_CT_). C. Deuterium incorporation spectra of peptide 365–391 (202_CT_-228_CT_) sequence NIEGRPTLSGIVSWGRGCAEKNKPGVY, in the +5 charge state. The *x*-axis of each plot is *m*/*z* and the *y* axis is the intensity. Each column represents one muPA variant (WT, no light chain, F(−2)A, K4CTG) and each row a deuteration time point (0–5 min). The envelope of the higher *m*/*z* conformation is shown in blue and the one of the lower *m*/*z*, in red.

The S1 specificity pocket is the binding site for the P1 residue of the substrate. The crystal structure of muPA^8^ shows that the specificity pocket is made up of D352 (189_CT_) in the 180s loop, and G379 (216_CT_) and G389 (226_CT_) in the 220s loop (Figure 6 A, spheres), which correspond to a trypsin-like specificity that would favor cleaving after R and K. Our HDX-MS results show that a peptide including the turn between β10 and β11, the entire β11 strand and the 220s loop (Figure 6C, S5 and S6) showed a bimodal deuterium uptake distribution with a more abundant lower *m*/*z* state in the presence of the light chain. In the absence of the light chain, this region is exchanging much more.

Interestingly, the F(−2_CT_)A and K4_CT_G mutants also had more of the highly exchanging state than the WT. Our results suggest that the light chain interacts with the turn between β10 and β11 and this interaction may affect the positioning of the residues of the specificity pocket in the 220s loop. The less dynamic the region, the more formed the specificity pocket would be.

### Effect of the Light Chain on the 140s Loop

**Figure 7:**
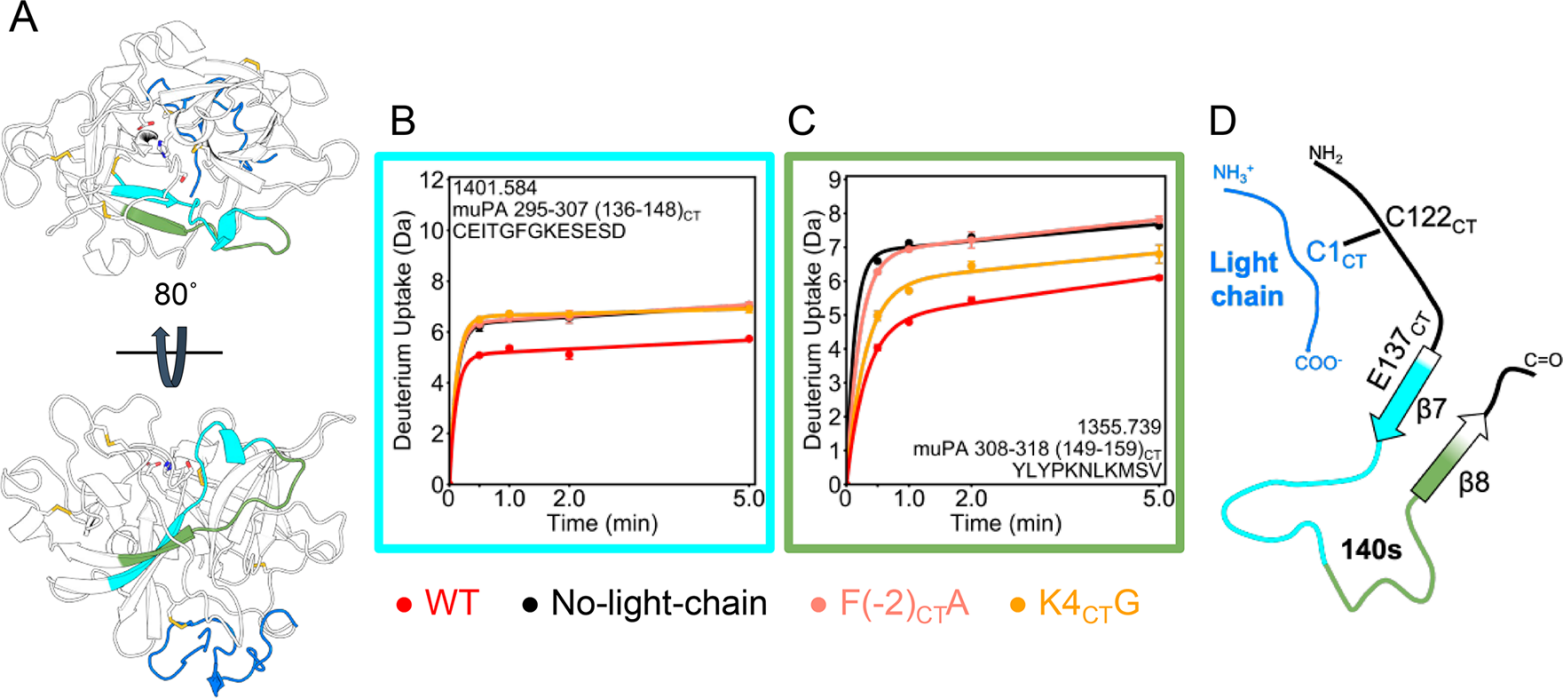
Light chain regulates the dynamics of the 140s loop. A. The 140s loop (colored in cyan and green) is at the “bottom” of the protease as seen from the canonical view (top). A peptide corresponding to residues 295–307 (136_CT_-148_CT_) representing the β7 strand and the beginning of the 140s loop is colored in cyan and a peptide corresponding to residues 308–318 (149_CT_-159_CT_) representing the last part of the 140s loop and part of the β8 strand is colored in green. B. Deuterium uptake plot of residues 295–307 (136_CT_-148_CT_) in the 140s loop. C. Deuterium uptake plot of residues 308–318 (149_CT_-159_CT_) in the 140s loop. D. Cartoon model showing where the light chain would interact to affect the 140s loop.

The most dramatic effect of the presence of the light chain occurred in the 140s loop (Figure 7). The first part of the 140s loop showed decreased exchange only when the WT light chain was present (Figure 7 B). Both mutants (K4_CT_G and F(−2_CT_)A) gave results equivalent to the No-light-chain protein. This region includes E296 (137_CT_) that was shown in the crystal structure of huPA to interact with K4_CT_ in the light chain.^17^ Interestingly, our AMD simulation suggested that E296 (137_CT_) could be interacting with three other residues in the light chain: R155 (7_CT_), R157 (9_CT_) and K159 (15_CT_) (Figure S7). The latter part of the 140s loop showed a dramatic decrease in exchange in the presence of the WT light chain (Figure 7 C). The F(−2_CT_)A showed the same level of deuterium uptake as the No-light-chain condition, whereas the K4_CT_G exhibited a deuterium exchange level in between that of the WT and the No-light-chain muPA. Strikingly, this trend is inversely proportional to the amidolytic activity of each protein (Table 1).

### Effect of the Light Chain Mutants on the Protease Domain

**Figure 8:**
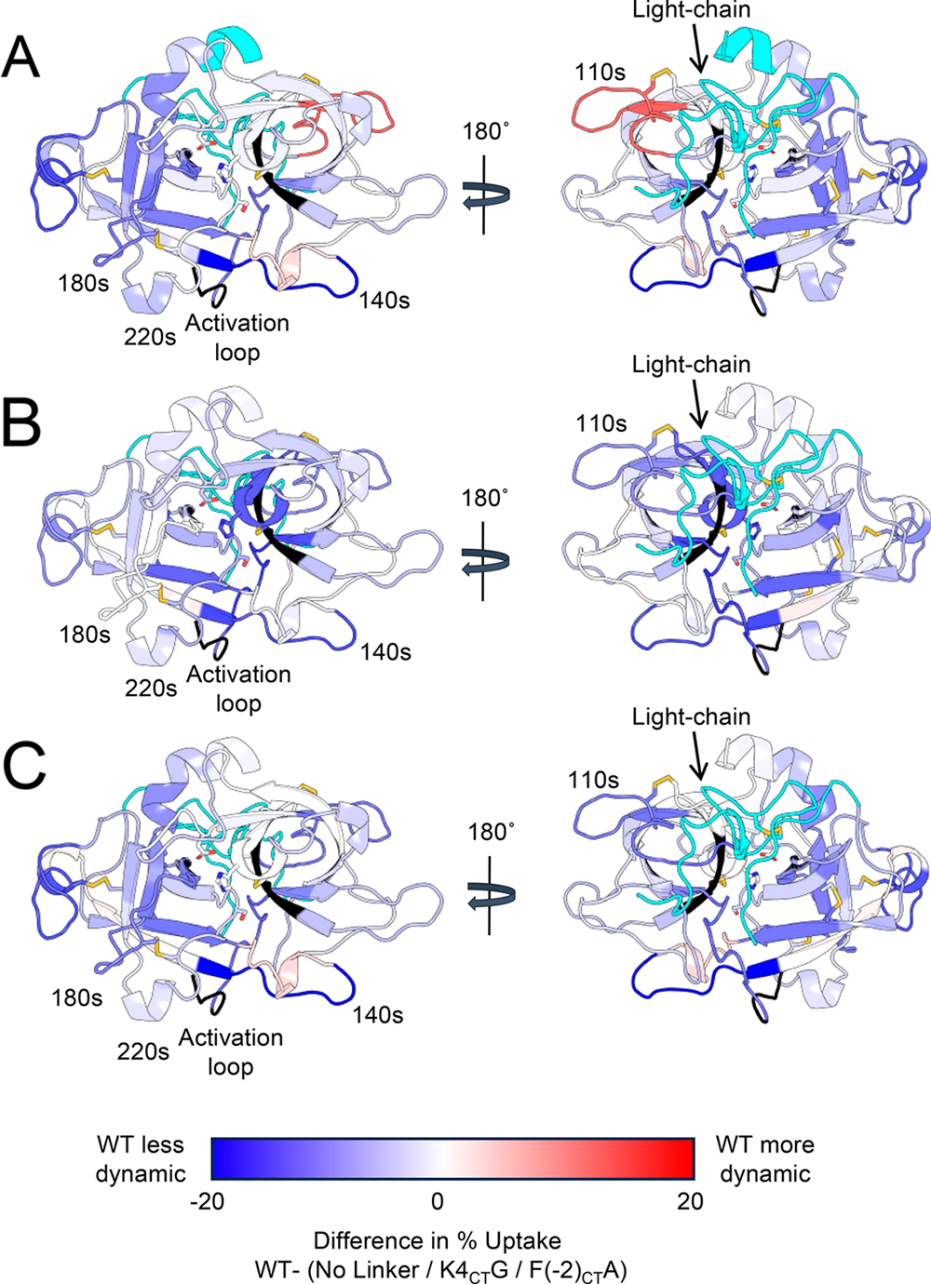
Summary of the HDX-MS changes in the protease domain due to the light-chain and the mutants K4_CT_G and F(−2_CT_)A The difference of deuterium uptake between WT and the protease lacking the light chain (A) or the K4_CT_G mutant (B) or the F(−2_CT_)A mutant (C) were mapped onto a conformation of the ensemble acquired by muPA WT during the AMD. The peptides are colored blue if the WT is less dynamic or red if its more dynamic that the compared condition. The regions that were not covered in the HDX-MS are shown in black. The regions that were covered only in one condition, or that had a different mutation in each condition and are not directly comparable, are shown in cyan.

To explore the influence of the light chain on the conformational dynamics of the muPA protease domain considering the protein as a whole and not as individual parts, we mapped the differences in percent deuterium uptake between the No-light-chain protein or the mutants vs WT onto a conformation of the ensemble of muPA WT from the AMD (Figure 8). Our results show that the light chain influences the dynamics of the protease domain mostly by altering the C-terminal β-barrel, with the most dramatic effects seen in the 140s loop (Figure 8 A). The K4_CT_G mutation affected the 140s loop and parts of the N-terminal β-barrel, including the loop where the catalytic H206 (57_CT_) is located. The F(−2_CT_)A mutation affected the C-terminal β-barrel more broadly, and more strongly resembled the effects of removal of the light chain all together. Our results suggest that the light chain interacts with the “back side” of the protease, opposite to where the substrate would enter into the specificity pocket, and that this interaction is transmitted to the active site of the protease, affecting the catalytic triad.

## Discussion

The fact that the amides of the disordered part of light chain are all highly exchanging suggests that this region is highly dynamic and without any stable secondary structure. AMD showed that the light chain, which is tethered by the disulfide bond to the protease domain is otherwise highly disordered. How, then, does it affect the deuterium exchange of the rest of the protease domain? As in “fuzzy” complexes,^35^ the presence of the light chain likely forms transient interactions with nearby loops, which then transmit changes in dynamics to other parts of the protein.

**Figure 9:**
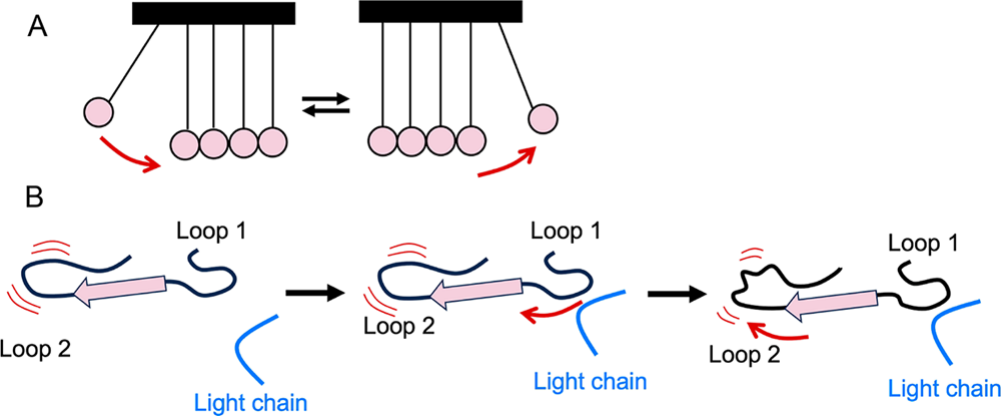
Model of how dynamic allostery caused by the light chain interactions is transmitted through the β-strands between loops. A. Dynamic allostery works as a Newton’s cradle, where changes in one side of the device are transmitted through the middle. The interactions of the middle balls do not change, and we can only observe the transmission of energy on the balls in the extremes. B. In the absence of the light chain, loops 1 and 2 are dynamic and connected covalently through a β-strand. When the light chain interacts with loop 1, that energy is transmitted to loop 2, which changes its dynamics and hydrogen bonding patterns. As the light chain is dynamic, it can also move away and stop interacting with loop 1, which returns its dynamics to the original conformation. The lack of interaction is also transmitted through the β-strand and returns the dynamics of loop 2 to its original state as well. The hydrogen bonding network and solvent accessibility of the amides in the β-strand does not change during this process.

The changes in deuterium exchange observed in regions that are not expected to be directly in contact with the light chain and seem to be transmitted through the β-strands are an example of how dynamic allostery is represented by a Newton’s cradle (Figure 9).^36^ In a Newton’s cradle, the force applied to one side gets transmitted to the other side without the middle apparently being affected (Figure 9 A). Our results show interaction of the light chain with the β10- β11 turn is transmitted through β11 to the 220s loop and specificity pocket and interaction of the light chain with the β7 strand affects the 140s loop (Figure 9 B). Note that the β-strand connectivity, solvent accessibility, or hydrogen bonding pattern are not altered in this mechanism, which is why it is not possible to measure the β-strand motions by HDX-MS. However, other techniques like NMR can measure the β-strand dynamics, as has been shown previously for the β-strands of the serine protein thrombin when binding thrombomodulin.^34,36^

Strikingly, we show that the light chain of muPA dampens the dynamics of the 140s loop. Two light chain mutations that partially or completely disrupted the interactions between the light chain and the protease domain had increased deuterium exchange in the 140s loop either partially or completely. The light chain interacts with the β7 strand that leads into the 140s loop and most likely dampens its dynamics which then allosterically affect the ability of the new N-terminus to stably form the salt bridge that organizes the catalytic triad. This assertion is backed-up by the remarkable correlation we observed between the degree of decreased deuterium uptake and the improvement in *k*_cat_. We observed something similar in thrombin upon thrombomodulin binding. The most significant effect of thrombomodulin binding on thrombin was to dampen the dynamics of the 140s loop.^37^ The light chain of thrombin and muPA only have 2 conserved residues (C1_CT_ and G2_CT_). Molecular dynamics simulations have predicted that mutation of R4_CT_A in thrombin would affect the dynamics of the heavy chain of thrombin, far beyond the points of contact with the light chain.^38,39^ The thrombin R4_CT_A and the muPA K4_CT_ both interact with E137_CT_. In thrombin, this mutation affected the conformation of the catalytic triad, and the dynamics of the 170s, 180s and 220s loops in thrombin.^38^ Our HDX-MS data shows mutation of the K4_CT_G in muPA affects the 140s loop, the activation loop, and the 220s loop. Thus, the light chain of both thrombin and muPA allosterically alter both β-barrels of the serine protease and their catalytic activity. Interestingly, crystal structures of the serine protease tissue-type plasminogen activator from human and bats also show the interaction between R4_CT_ and E137_CT_,^40,41^ and these residues together with F(−2)_CT_ are highly conserved in uPA from different species.^17^ This evidence suggests that the activity of other serine proteases or of other uPA orthologues could also be allosterically regulated by the interactions with their light chains.

Kromann-Hansen et. al previously showed that the presence of an active site inhibitor, Glu-Gly-Arg chloromethylketone, would decrease deuterium uptake in some regions of the protease domain of muPA in the absence of the light chain, including the activation loop, the 140s, the 180s, the 220s and the β9 strand.^10^ Addition of the light chain was enough to dampen deuterium uptake levels of the 180s loop, part of the 140s loop (residues 136_CT_-148_CT_) and of the β9 strand to the same degree as binding of the inhibitor. However, the light chain by itself was insufficient to reduce deuterium uptake of the loops involved in catalysis, like the activation loop, part of the 140s (residues 149_CT_-159_CT_), and the 220s loop. We expect that the addition of the inhibitor to the protease domain in the presence of the light chain would decrease the deuterium uptake of these loops to the same level as the reported previously in the absence of the light chain.

## Conclusion

Although the light-chain of muPA is highly disordered, it strongly enhances its catalytic activity. AMD and HDX-MS suggested that the dynamic light-chain of muPA dampens the dynamics of the loop after the activation loop, the turn before the 220s loop and the β-strand before the 140s loop. We propose that this reduced conformational ensemble more closely resembles the active chymotrypsin-like fold than the inactive nonchymotrypsin-like fold previously crystallized.^8^ Two light-chain mutations that partially or fully decreased the protease activity of muPA: K4_CT_G and F(−2_CT_)A with correlated partial increases in dynamics of the 140s and 220s loops bolstered the assertion that loop conformations correlate with protease activity. Finally, we proposed a model for the observed allosteric effects of the light chain on catalytic activity in which the β-strands forming the C-terminal β-barrel could transmit the energy from the interaction between the light-chain and the protease domain to the loops on the opposite side of the protease domain, just like the spheres in a Newton’s cradle can transmit the energy from one side to the other.

## Data Availability

HDXMS data is publicly available at massive.ucsd.edu data set MSV000094027. Structures are available on Zenodo at 10.5281/zenodo.10627181.

## Supporting Information Available

Additional figures: Michaelis–Menten curves of activity assays, additional HDX-MS plots and spectra, conformations of the light chain showing additional interaction with E296 (137_CT_); a table of HDX-MS parameters (PDF)
