# Supplementary material for: The Light Chain Allosterically Enhances the Protease Activity of Murine Urokinase-Type Plasminogen Activator
Source: Biochemistry. 2024 May 23;63(11):1434–44. doi: 10.1021/acs.biochem.4c00071 (PMC11154964; doi:10.1021/acs.biochem.4c00071)
Supplement: Supplementary file 1 — bi4c00071_si_001.pdf [file bi4c00071_si_001.pdf]

Supporting Information

The light chain allosterically enhances the protease activity of murine urokinase-type plasminogen activator

*Constanza Torres-Paris[1], Harriet J. Song[1], Felipe Engelberger[2,3], César A. Ramírez-Sarmiento[2,3], Elizabeth A. Komives[1]**

AUTHOR ADDRESS. [1] Department of Chemistry and Biochemistry, Mail Code 0309, University of California, San Diego, 9325 S Scholars Dr, La Jolla, CA 92161

[2] Institute for Biological and Medical Engineering, Schools of Engineering, Medicine and Biological Sciences, Pontificia Universidad Católica de Chile, Santiago 7820436, Chile.

[3] ANID - Millennium Science Initiative Program - Millennium Institute for Integrative Biology (iBio), Santiago 8331150, Chile.

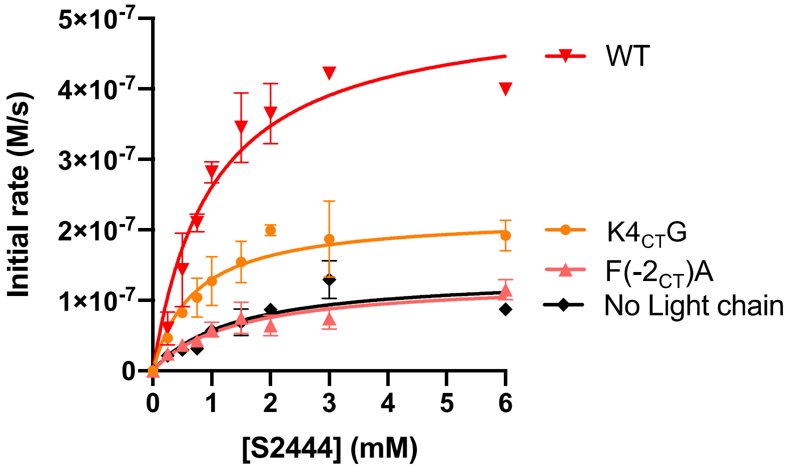

Figure S1. Michaelis-Menten plots of the hydrolysis of S2444 catalyzed by 10 nM muPA WT, $K4_{CT}G$, $F(-2_{CT}A)$ or No-light-chain. The reactions were monitored for 10 min at 37˚C. Each data point represents the average of two technical replicates and its standard deviation.

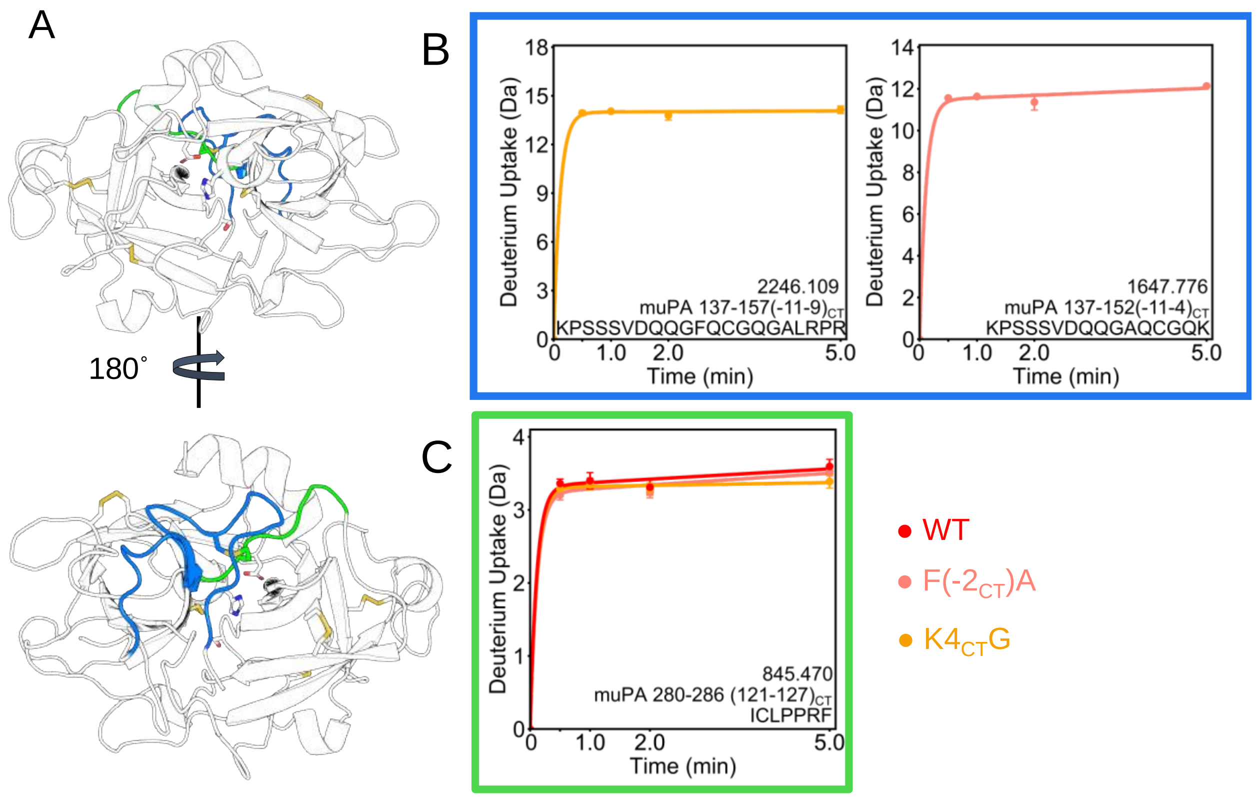

Figure S2. The light chain in the muPA mutants K4$_{CT}$G and F(-2$_{CT}$)A is very dynamic. A. Canonical (top) and rotated (bottom) view of a conformation of the ensemble acquired by muPA WT in AMD. The catalytic triad residues and the disulfide bonds are shown as sticks. The region spanning residues from the light chain are colored in blue and residues 280-286 (121$_{CT}$ -127$_{CT}$) are colored in green. These representations are the same as in Figure 2C. B. Deuterium uptake plots of the light chain peptides of muPA K4$_{CT}$G (left) and F(-2$_{CT}$)A (right). Each data point represents the average of three technical replicates and the error bars represent the standard deviation. C. Deuterium uptake plots of the protease peptides which contain C282 (C122$_{CT}$), the site where the light chain covalently binds to the protease.

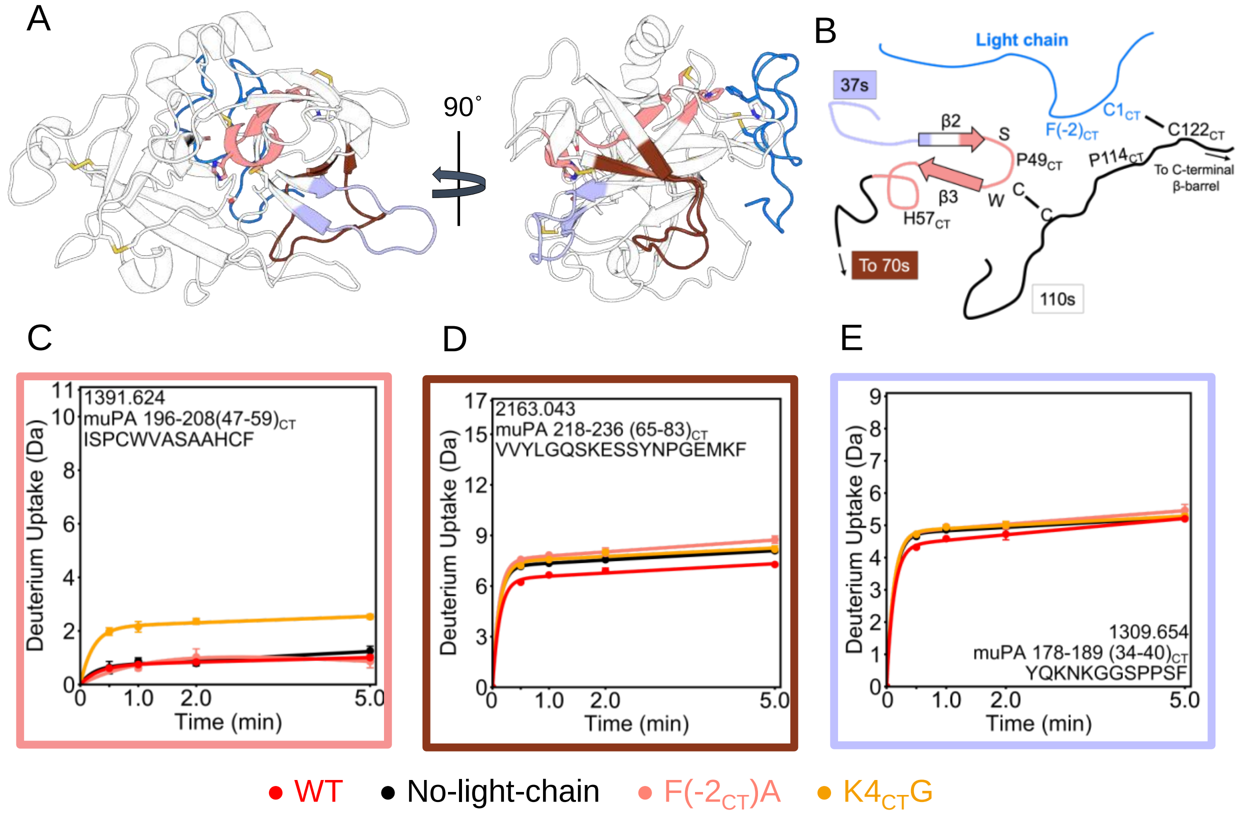

Figure S3. Effect of the light chain on the N-terminal β-barrel. A. Canonical (right) and rotated (left) view of a conformational of the ensemble acquired by muPA WT during the AMD. A peptide spanning the turn between β2 and β3 and the entire β3 is shown in pink (residues 196-208 (47_{CT}-59_{CT})), the 70s loop is shown in brown (residues 218-236 (65_{CT}-83_{CT})) and the 37s loop is shown in light violet (residues 178-189 (34_{CT}-40_{CT})). The light chain is colored in blue. B. Cartoon representation of the interactions between the light chain and the N-terminal β-barrel. C. Deuterium uptake plot of residues 196-208 (47_{CT}-59_{CT}) covering H206 (57_{CT}). D. Deuterium uptake plot of residues 218-236 (65_{CT}-83_{CT}) in the 70s loop. E. Deuterium uptake plot of residues 178-189 (34_{CT}-40_{CT}) in the 37s loop.

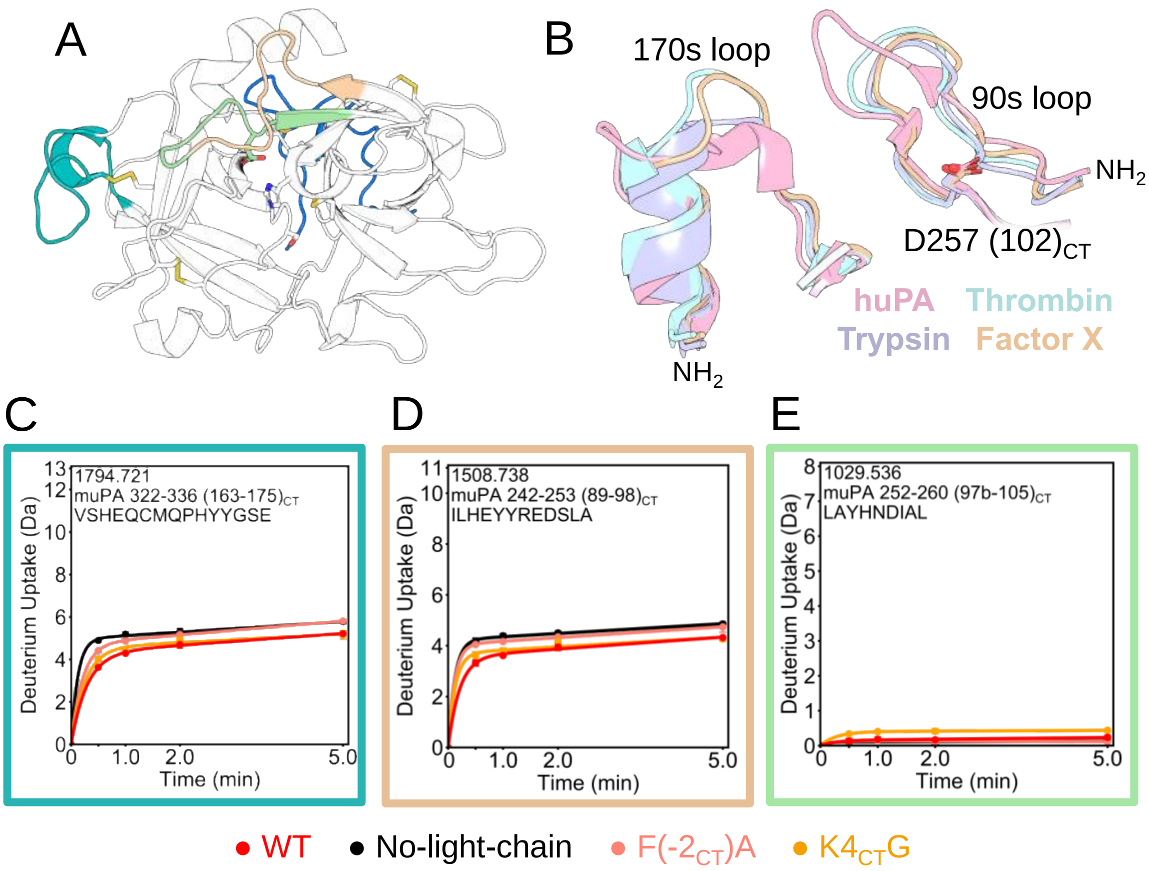

Figure S4. The light chain enhances the interactions between the 170s and the 90s loop, which is longer in uPA than in other serine proteases. A. The 170s loop (residues 322-336 (163_{CT}-175_{CT}), teal) is near the 90s loop in the conformation acquired by muPA WT in AMD. The first portion of the 90s loop (242-253(89_{CT}-98_{CT})) is colored in cream and its second portion (252-260($97b_{CT}$-105_{CT})) is in light green. This last peptide encompasses the catalytic aspartic acid D257(102_{CT}). B. Structural alignment of the 90s and 170s loops of four serine proteases: huPA (light pink, PDB: 1O3P), trypsin (light purple, PDB: 1C5P), thrombin (light blue, PDB:1C5L) and Factor X (light orange, PDB: 1C5M). The catalytic $D102_{CT}$ are shown as sticks. C. Deuterium uptake plot of residues 322-336 (163_{CT}-175_{CT}) 170s loop. D. Deuterium uptake plot of residues 242-253 (89_{CT}-98_{CT}) in the 90s loop. E. Deuterium uptake plot of residues 252-260 ($97b_{CT}$-105_{CT}) corresponding to the latter portion of the 90s loop. This last region contains the catalytic D257 (102_{CT}).

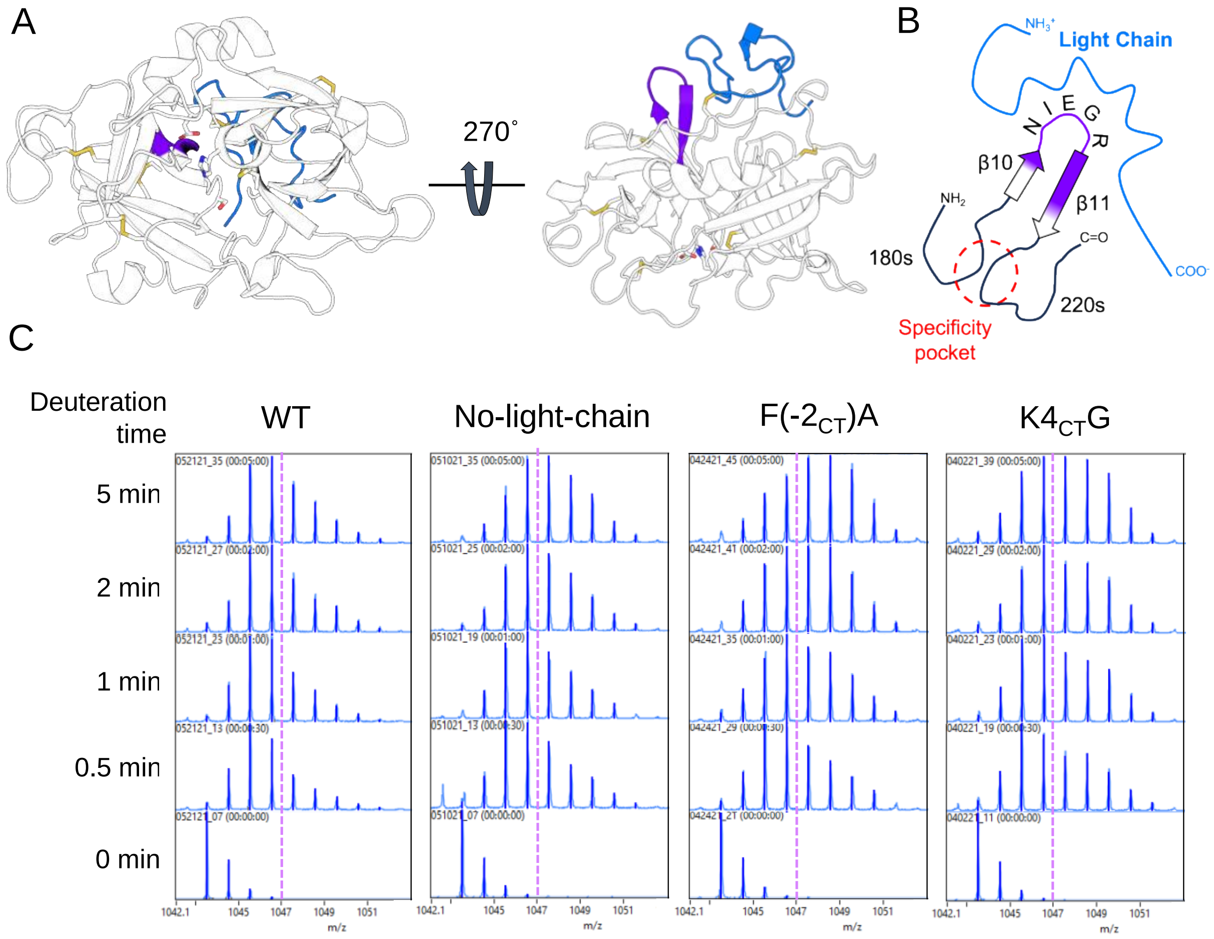

Figure S5. The light chain affects the dynamics of the β10-β11 turn. A. The β10-β11 turn (colored in purple) is in the C-terminal β-barrel of muPA WT, as shown by this conformation in the AMD ensemble of muPA WT. The structure in the left represents the canonical view of the protein, and the structure in the right shows a 270˚ rotation on the x axis of the protease domain compared to the canonical view. The light chain is shown in blue. B. Cartoon model showing how the light chain would interact with the region covering residues 365-374 (202_{CT}-211_{CT}). C. Deuterium incorporation spectra of peptide 365-374 (202_{CT}-211_{CT}) sequence NIEGRPTLSG. The x-axis of each plot is m/z and the y axis is the intensity of the peptides detected at each m/z. Each column represents one muPA variant (WT, No-light-chain, F(-2_{CT})A, $K4_{CT}G$) and each row a deuteration time point (0-5 min).

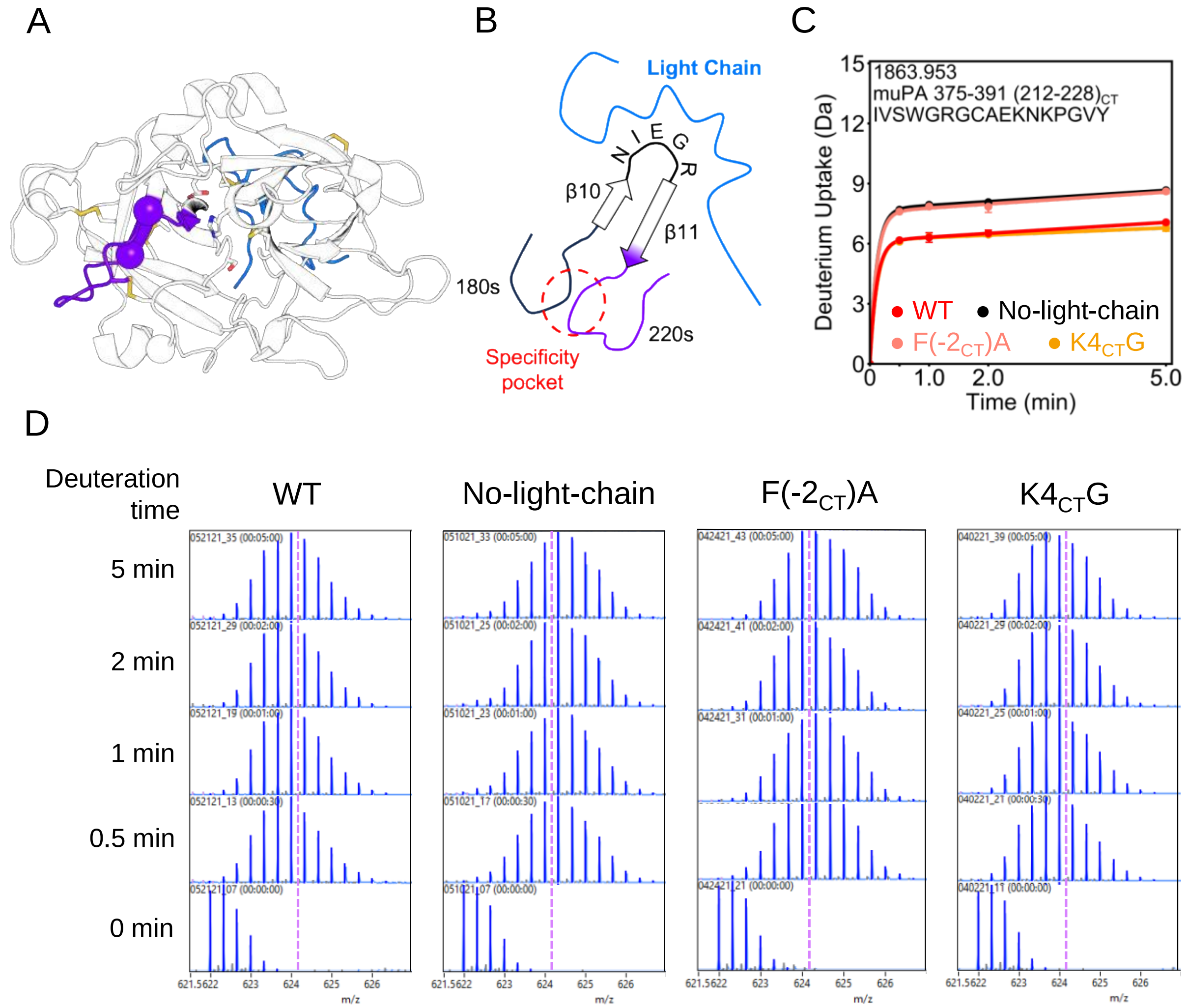

Figure S6. The light chain affects the dynamics of the 220s loop. A. The 220s loop (colored in purple) is in the C-terminal β-barrel of muPA WT, as shown by this conformation in the ensemble of muPA WT. The S1 specificity pocket is formed by two residues in the 220s loop (Gly379 (216_{CT}) and Gly389 (226_{CT}), shown as purple spheres) and a residue in the 180s loop (Asp352 (189_{CT}), shown as a white sphere. The light-chain is shown in blue. B. Cartoon model showing how the light chain would interact with the region covering residues 375-391 (212_{CT}-228_{CT}). C. Deuterium uptake plot of residues 375-391 (212_{CT}-228_{CT}). D. Deuterium incorporation spectra of peptide 375-391 (212_{CT}-228_{CT}) sequence IVSWGRGCAEKNKPGVY. The x-axis of each plot is m/z and the y axis is the intensity of the peptides detected at each m/z. Each column represents one muPA variant (WT, No-light-chain, F(-2_{CT})A, $K4_{CT}G$) and each row a deuteration time point (0-5 min).

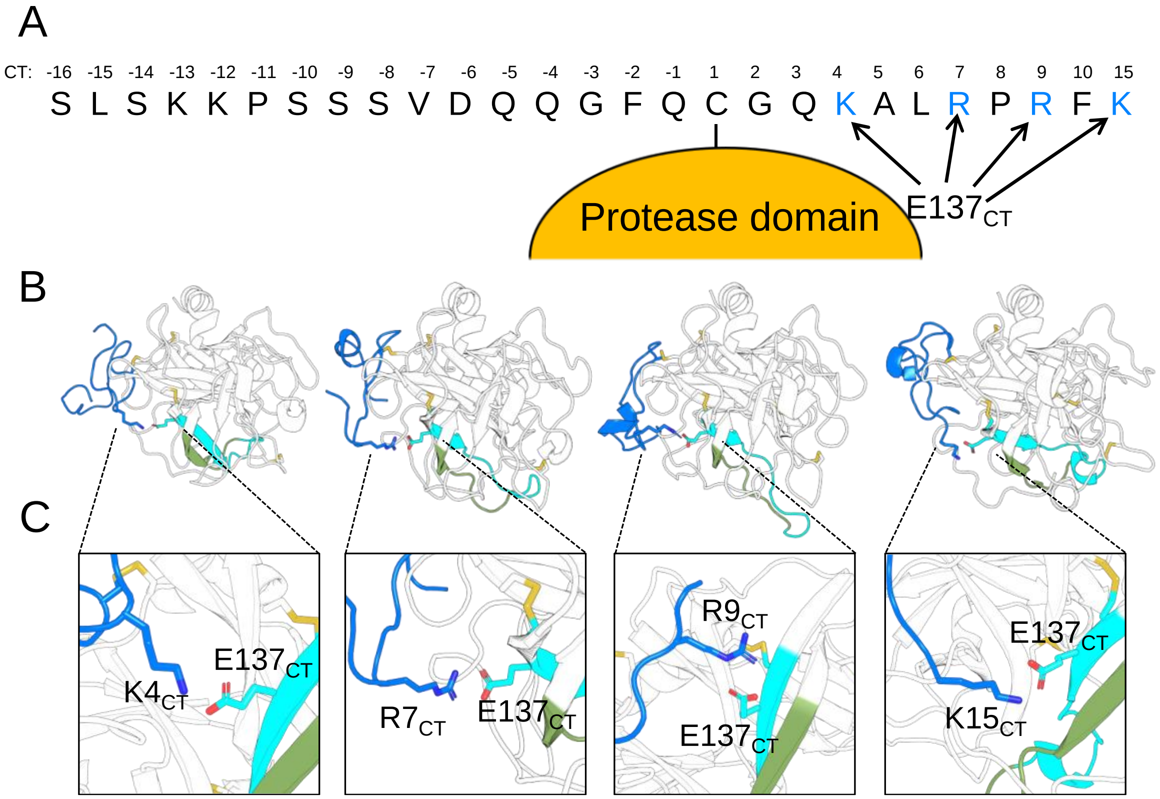

Figure S7. AMD shows that E296 (137_{CT}) can interact with K152 (4_{CT}), R155 (7_{CT}), R157 (9_{CT}) and K159 (15_{CT}) in the light chain. A. Sequence of the light chain of muPA WT. The residues of the light chain that interact with E296 (137_{CT}) are highlighted in blue. B. Examples of four conformations of the ensemble of muPA WT showing the four possible salt bridges formed by E296 (137_{CT}) and the light chain. C. Close-up of the conformations in B.

Table S1. HDX-MS parameters

**Data set (muPA)**	**WT**	**No Light Chain**	**F(-2$_{CT}$)A**	**K4$_{CT}$G**
HDX reaction details	10 mM Na_2HPO_4, 1.8 mM KH_2PO_4, 2.7 mM KCl, 137 mM NaCl (PBS), pH 7.4, 22 °C			
HDX time course (min)	0.5, 1, 2, 5			
HDX control samples	We use the unstructured ends of the protein as maximally deuterated controls.			
Back-exchange (mean / IQR)	28 % / 2 %	28 % / 2 %	28 % / 2 %	28 % / 2 %
# of Peptides	113	85	110	113
Sequence coverage	95%	95%	92%	95%
Average peptide length / Redundancy	12.9 / 5.2	12.4 / 3.9	12.7 / 5.0	12.9 / 5.2
Replicates (biological or technical)	3 technical replicates each			
Repeatability	0.049 (average standard deviation)	0.048 (average standard deviation)	0.068 (average standard deviation)	0.056 (average standard deviation)
Significant differences in HDX (delta HDX > X D)	0.5 D (99% CI)			
